# Supplementary material for: Beyond the Interface: Improved Pulmonary Surfactant-Assisted Drug Delivery through Surface-Associated Structures
Source: Pharmaceutics. 2023 Jan 11;15(1):256. doi: 10.3390/pharmaceutics15010256 (PMC9866215; doi:10.3390/pharmaceutics15010256)
Supplement: Supplementary file 1 [file pharmaceutics-15-00256-s001.zip › pharmaceutics-2102293-supplementary.pdf]

Supplementary methods

Synthesis of the Red-Fluorescent NBA-Tagged Budesonide (F-BUD)

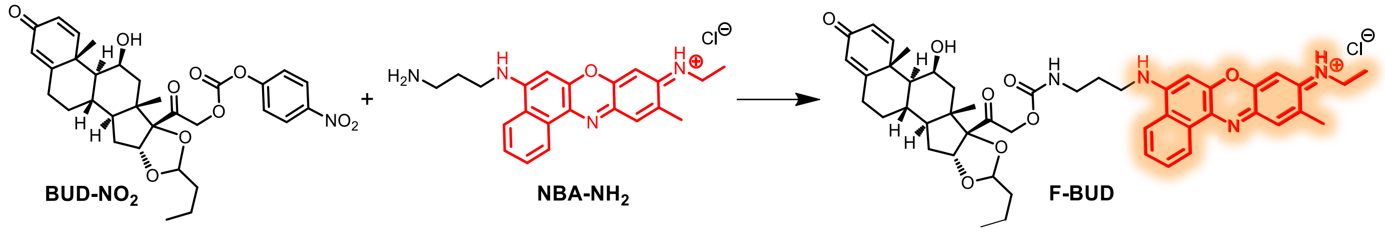

Scheme S1. Chemical structure of the reagents used to produce the red-fluorescent-labelled budesonide (F-BUD).

General methods

All chemicals were purchased from commercial suppliers (Sigma-Aldrich, Acros Organics, Fluorochem, Alfa Aesar or Carbosynth, except otherwise stated) and used without further purification. Water was from a Merck-Millipore Direct-Q3 purification system. Thin-layer chromatography (TLC) was carried out on pre-coated silica gel 60 F_{254} plates (Merck) with detection under UV light (254 or 365 nm). ^{1}H- and ^{13}C-NMR spectra were recorded at the UCM Research Support Centre (CAI) for NMR, on a Bruker AVIII 500 spectrometer operating at 500 MHz (125 MHz for ^{13}C), with deuterated chloroform as solvent. Chemical shifts are reported in ppm from TMS on the δ scale. In the case of multiplets, the signals are reported as intervals. Signals are abbreviated as s, singlet; d, doublet; t, triplet; quin, quintuplet, and m, multiplet. Coupling constants are expressed in Hz.

Synthesis of the red-fluorescent Nile Blue Analogue-labelled Budesonide (F-BUD)

Prepared by following the procedure of Kern et al. [48]. To a solution of budesonide-NO_2[1] (20 mg, 0.033 mmol) and anhydrous triethylamine (5.6 mL, 0.040 mmol) in anhydrous CH_2Cl_2 (1 mL), NBA-NH_2[2] (16 mg, 0.040 mmol) in anhydrous methanol (1 mL) was added (**Scheme S1**). The synthesis of the amino-derivative of the Nile Blue analogue, **NBA-NH$_2$**, has been already reported [20]. After stirring the mixture at room temperature for 72 h, a new blue spot was observed by TLC and NBA-NH_2 was no longer visible. The solvent was then evaporated under reduced pressure. The remaining solid was washed with hexane (3 x 10 mL), diethyl ether (3 x 10 mL), and ethyl acetate (3 x 10 mL). The resulting crude product was purified using silica gel column chromatography (eluent: CH_2Cl_2/MeOH, 99:1 to 90:10 v/v) to yield F-BUD as a pure blue solid (11 mg, 39%) (**Figure S1**). ESI-MS(+) 817.4 M^+; exact mass 817.42); ^{1}H-NMR (500 MHz) (δ/ppm): 11.01 (s, 1H), 9.06 (s, 1H), 8.74 (d, J = 8 Hz, 1H), 7.90-7.75 (m, 2H), 7.50 (m, 1H), 7.41 (d , J = 13.2 Hz, 1H), 6.55-6.43 (m, 1H), 6.26 (d , J = 13.6 Hz, 1H) , 6.01 (s, 1H), 5.17-5.09 (m, 2H), 4.80-4.68 (m, 2H), 4.65-4.45 (m, 2H), 3.82 (2, 2H), 3.43-3.36 (m, 3H), 2.60- 2.19 (m, 10H), 2.05-1.99 (m, 4H), 1.72-1.48 (m, 7H), 1.39-1.25 (m, 6H), 1.10-0.82 (m, 10H). ^{13}C-NMR (125 MHz) (δ/ppm): 186.8 (CO), 176.5 (CO), 170.5 (C), 166.2 (C), 156.4 (CH), 155.0 (C), 153.2 (C), 151.0 (C), 146.8 (C), 133.5 (CH), 130.8 (CH), 130.7 (C), 129.2 (CH), 127.7 (CH), 127.6 (C), 125.5 (CH), 122.8 (CH), 122.4 (CH), 93.8 (CH), 92.9 (CH), 87.9 (C), 83.8 (C), 75.6 (CH), 49.9, 48.7, 48.2, 48.0, 47.8, 47.6, 47.4, 47.1, 46.4, 46.1, 44.3, 42.0, 38.9, 37.1, 36.1, 35.1, 32.0, 31.2, 26.7, 21.0, 17.7, 17.5, 17.1, 14.1, 14.0.

Acquisition Parameter

Ion Source Type	ESI	Ion Polarity	Positive	Alternating Ion Polarity	off
Mass Range Mode	Ultra Scan	Scan Begin	100 m/z	Scan End	1400 m/z
Capillary Exit	160.2 Volt	Skimmer	40.0 Volt	Trap Drive	92.3
Accumulation Time	69 µs	Averages	7 Spectra	Auto MS/MS	off

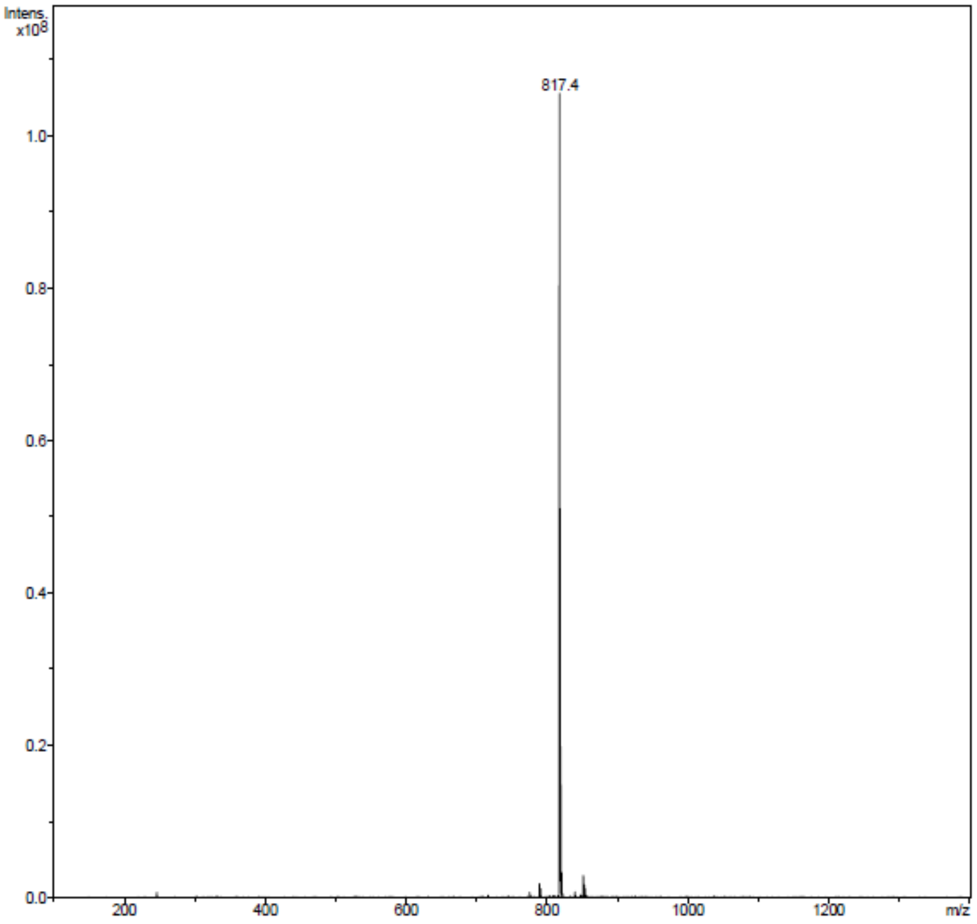

Figure S1. Mass spectrum (ESI, positive ions detection) of the pseudo Nile Blue-labelled Budesonide (F-BUD).

Supplementary figure

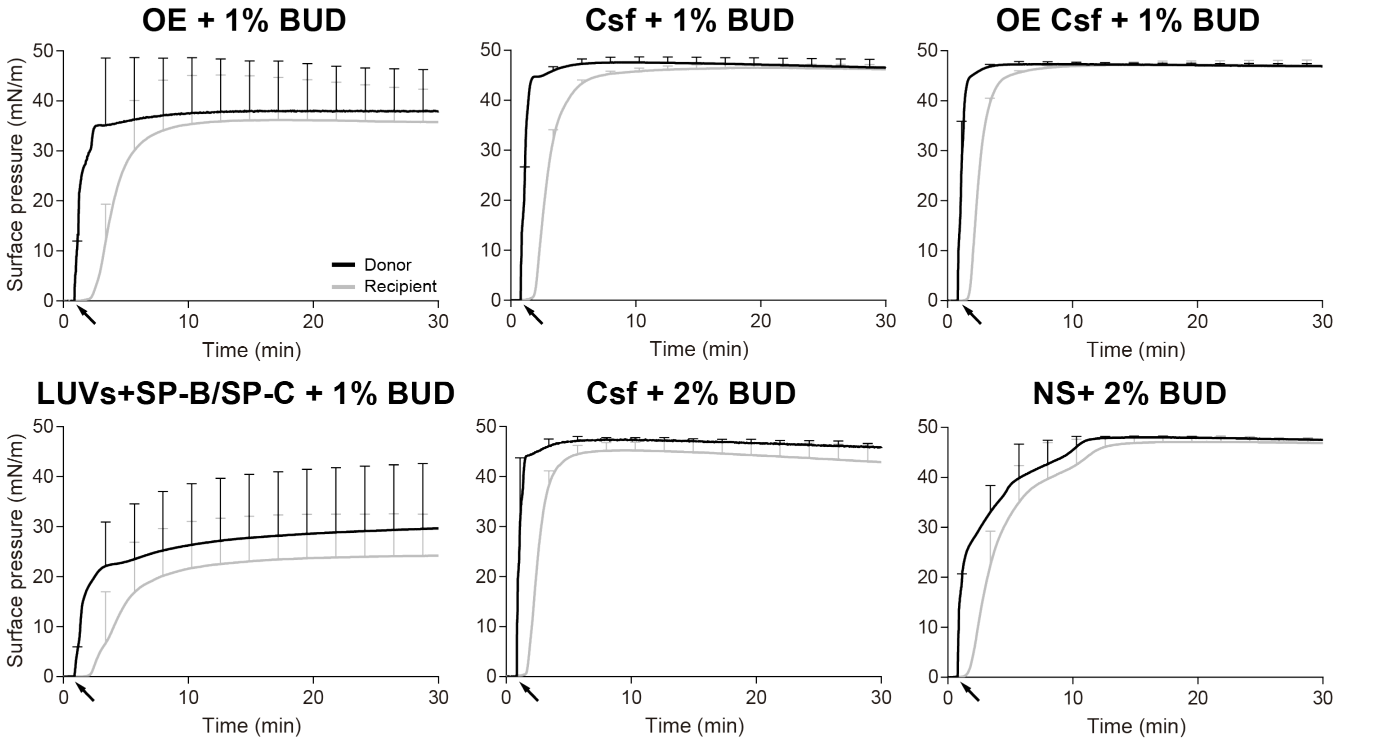

Figure S2. Vehiculization of Budesonide by different pulmonary surfactant formulations in the presence of the paper bridge. Adsorption/spreading isotherms derived from experiments performed in the presence of the bridge, upon the injection of 15 μL (20 mg/mL) of aqueous suspension of OE (top left), Csf (top middle), aqueous suspension of an OE of Csf (top right) and proteo-lipid LUVs (bottom left) combined with 1% (w/w with respect to total mass of phospholipids) BUD, and Csf (bottom middle) and NS (bottom right) in combination with 2% (w/w) BUD.

References

20. Hidalgo, A., Salomone, F., Fresno, N., Orellana, G., Cruz, A., Perez-Gil, J. Efficient interfacially driven vehiculization of corticosteroids by pulmonary surfactant. *Langmuir* **2017**, *33* (32), pp. 7929-7939.

48. Kern, J.C., Dooney, D., Zhang, R., Liang, L., Brandish, P.E., Cheng, M., Feng, G., Beck, A., Bresson, D., Firdos, J. Novel phosphate modified cathepsin B linkers: improving aqueous solubility and enhancing payload scope of ADCs. *Bioconjug. Chem.* **2016**, *27* (9), pp. 2081-2088.
